# Classification of endoscopic spine procedures

**DOI:** 10.1016/j.xnsj.2025.100603

**Published:** 2025-03-11

**Authors:** Mazda Farshad, Christoph J. Laux, Florian Wanivenhaus, José M. Spirig, Jonas Widmer, Michael Kelly, Javier Quillo-Olvera, Jin-Sung Kim, Facundo van Isseldyk, Sohrab Gollogly, James Yue, Xuexiao Ma, Vincent Hagel, Frédéric Cornaz

**Affiliations:** a University Spine Center Zurich, Balgrist University Hospital, University of Zurich, Zurich, Switzerland; b Spine Biomechanics, Balgrist University Hospital, University of Zurich, Zurich, Switzerland; c Hospital for Special Surgery, New York, New York, United States; d Hospital Spine Center- Neurological Surgery Department, Hospital Angeles Centro Sur, Queretaro City, Mexico; e Spine Center, Department of Neurosurgery, Seoul St. Mary's Hospital, College of Medicine, The Catholic University of Korea, Seoul, South Korea; f National University of Rosario (UNR), Hospital Privado de Rosario (HPR) Rosario, Argentina; g Department of Surgery, Monterey Spine and Joint Center, Monterey, California, United States; h Orthopaedic Surgery, Frank H Netter School of Medicine, CT Orthopaedics, Hamden, Connecticut, United States; i Affiliated Hospital of Qingdao University, Qingdao, China; j Spine Center, Asklepios Hospital Lindau, Lindau, Germany

**Keywords:** Endoscopy, Complexity, EndoSpine, Classification, Consensus, Nomenclature

## Abstract

**Background:**

A consensus on grading the complexity of endoscopic spinal procedures is lacking, but urgently needed to guide training, clinical practice, and regulatory concepts.

**Methods:**

A 2-dimensional classification system was developed, considering both the technical and morphological parameters contributing to the complexity of endoscopic spine procedures. An international survey with 68 questions - including those on demographic data and surgical volumes, suitability of the proposed 2-dimensional classification system, and categories of techniques and morphologies - was completed by spine surgeons with endoscopic experience. A consensus was defined as a difference >/= 10% between the most frequently given grade and the second most given grade. In cases of no clear consensus (ie, agreement of less than 10%), an additional analysis considering only the responses from surgeons with experience of more than 500 endoscopic spine surgeries was performed.

**Results:**

115 survey entries were received, of which 112 were analyzed. The participating spine surgeons (64% orthopedic surgeons, 35% neurosurgeons, 1% other) originated from 27 countries and have performed an average of 509 endoscopic spine surgeries (55,984 total endoscopic procedures). 85.7% of the survey respondents agreed that the proposed 2-dimensional classification system was indeed appropriate for its particular purpose. Thus, a consensus classification system was born, allowing for grading simple procedures (eg, Ia for lumbar interlaminar discectomy of a soft disc herniation) to complex procedures (eg, IIIc for revision posterior endoscopic cervical central decompression).

**Conclusions:**

A consensus of 112 endoscopic spine surgeons from 27 countries facilitated the development of a 2-dimensional classification system outlining the complexity of endoscopic spinal procedures, taking into account both technical aspects and morphological parameters. This classification system categorizes different endoscopic spine procedures and the pathologies they are employed to treat based on complexity, thus guiding the endoscopic spine community in medical training, patient education, and regulatory and reimbursement discussion.

## Introduction

Endoscopic spine surgery is an emerging alternative to conventional open/microsurgical spine surgery for selected indications [[1], [2], [3], [4]]. It boasts potential advantages including reduced rates of dural tears [5], surgical site infections [[6], [7], [8]], and mid-term conversions to fusion surgery [9]. Furthermore, greater cost-effectiveness has been described for the treatment of lumbar stenosis and disc herniation when comparing spinal endoscopy to standard microsurgical procedures, with shorter length of stay and lower associated costs [10].

Spinal endoscopy includes several techniques with different nomenclature used. A comprehensive, systematic overview has been compiled in an international consensus paper [11], resulting in a classification of nomenclature describing these techniques, however a categorization based on complexities of procedures remains lacking.

The complexity of endoscopic interventions can vary by a large degree, and is typically based on 2 main factors; the technique itself and the specific pathology being addressed. In the setting of a rather flat learning curve for endoscopic spine surgery [[12], [13], [14]], it is of utmost importance to know which grade of complexity is faced when selecting a specific technique for a specific pathology. For this reason, a categorization aid is needed. The aim of this study therefore was to develop a classification system for the complexity of endoscopic spine procedures, incorporating complexity of different technical and morphological characteristics.

## Material and methods

**Fig. 1:**
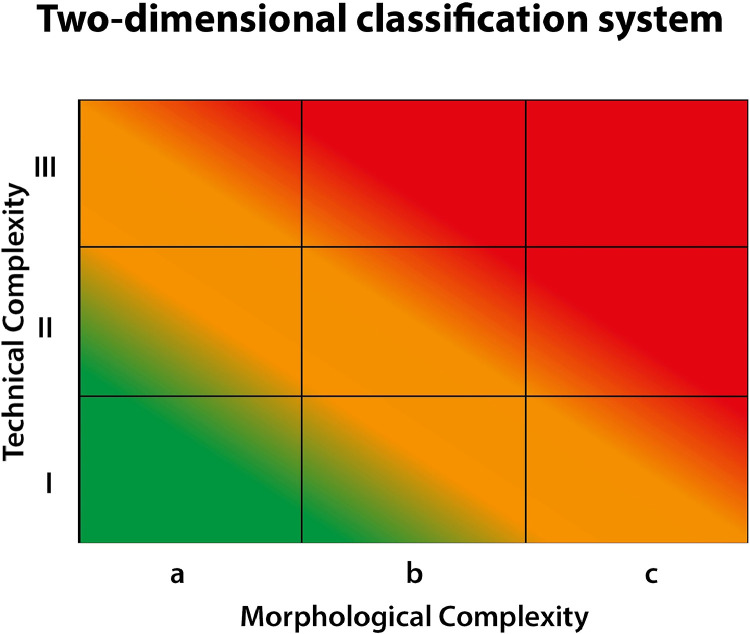
Two-dimensional classification to grade the complexity of endoscopic spine surgery.

First, a 2-dimensional classification system was developed as a grid to grade the complexity of endoscopic spine procedures (Fig. 1). The grid considers the technical (vertical, y axis) as well as the morphological complexity (horizontal, x axis) of endoscopic spine procedures. The technical complexity (grade I–III) describes the surgical technique/approach, while the morphological complexity (grade a–c) describes the anatomical and pathological conditions that may add complexity to the surgical procedure. By combining these 2 factors, a more nuanced evaluation of the complexity of each specific operation can be determined.

Based on the AOSpine nomenclature of endoscopic spine procedures [11], the authors proposed the following list of technical procedures: anterior endoscopic cervical discectomy (AECD), posterior endoscopic cervical foraminotomy (PECF), posterior endoscopic cervical central decompression (PECCD), thoracic endoscopic unilateral laminotomy for bilateral decompression (TE-ULBD), transforaminal thoracic decompression/discectomy (TETD), transforaminal endoscopic lumbar discectomy (TELD), interlaminar endoscopic lumbar discectomy (IELD), extraforaminal endoscopic lumbar discectomy (EELD), transforaminal endoscopic lateral recess decompression (TE-LRD), interlaminar endoscopic lateral recess decompression (IE-LRD), transforaminal endoscopic lumbar foraminotomy (TELF), interlaminar contralateral endoscopic lumbar foraminotomy (ICELF), lumbar endoscopic unilateral laminotomy for bilateral decompression (LE-ULBD), and endoscopic fusion (EF).

Regarding the morphological parameters, the following factors were proposed: easy access (eg, interlaminar L5/S1), spinal level adding difficulties (eg, transforaminal approach to L5/S1), potential danger to critical structures (eg, vertebral artery looping), soft disc herniation, relevant spondylolisthesis/deformity, severe degenerative changes, scarring (eg, revision at the ipsilateral side), calcified disc herniation, and osseous/osteodiscal stenosis.

To evaluate the utility of the proposed 2-dimensional classification system and to classify the technical and morphological parameters, an online survey composed of 68 questions was created on REDCap (Research Electronic Data Capture) and distributed amongst spine surgeons with endoscopic experience via direct contact, e-mail, or social media (eg, LinkedIn, https://www.linkedin.com). The survey first gathered information on demographic characteristics and surgical experience of the respondents. Then, the survey prompted an evaluation of the proposed 2-dimensional classification system, asking respondents to grade the complexity of the surgical techniques and morphological conditions, while allowing respondents to provide suggestions and critiques. Furthermore, it included questions on respondents’ opinions regarding the utility of endoscopic training methods (simulation and cadaveric training) as well as the number of simple cases that should be performed before advancing to more complex surgeries. The respondents were allowed to provide their personal information (name and e-mail address) and were asked whether they would like to be informed of the study results. The full survey is provided in the appendix (Appendix 1). The survey was published on September 05, 2024, and evaluation of the responses was performed on September 26, 2024. The survey data was ultimately analyzed using descriptive statistics utilizing Excel (Microsoft Excel 2019, Microsoft Corporation).

The grades of the technical and the morphological parameters were determined by the most frequent grade given by survey's respondents. A consensus grade was determined when the difference between the most frequently given grade and the second-most frequently given grade was larger than 10%. In cases of no clear consensus among the survey respondents (ie, agreement of less than 10%), an additional analysis considering only the responses from surgeons with experience of more than 500 endoscopic spine surgeries was performed.

## Results

The online survey was completed by 115 surgeons, of which 112 were included in the final evaluation: 106 surveys were complete, while 6 were largely complete (less than 10 missing entries). 3 surveys were largely empty (less than 10 total entries) and for this reason were ultimately excluded from the analysis.

The 112 included surveys were completed by respondents originating from 27 different countries, with the largest cadre of 34 surgeons from Mexico. 15 respondents were from the USA, 12 were from Switzerland, 6 were from India, and 4 each were from Spain, Brazil, and Germany. 44.5% of the respondents were from North America, 24.5% were from Europe, 15.6% were from Asia, 13.6% were from South America, and 0.9% were from Africa. 91 respondents (81%) provided their name and contact details. 72 respondents were orthopedic surgeons, 39 were neurosurgeons, and 1 respondent did not provide this information. 103 respondents were male, 7 were female, 1 defined their gender as other, and 1 respondent did not provide any information in this regard. The average age of the respondents was 45 years (range 31–84 years). 61 respondents work in the private sector, 21 work in a university hospital environment, and 27 work in the public sector. 25 respondents are employed as chief surgeons, 42 as consulting surgeons, and 39 are self-employed.

The respondents have been performing spine surgery (all techniques) for an average of 14.1 years and endoscopic spine surgery for an average of 5.9 years. Monoportal surgery is performed by 58 respondents, biportal by 20, both techniques by 30 surgeons and 4 surgeons did not provide this information. The respondents have performed an average of 509 endoscopic spine surgeries in their careers (median 150), totaling 55,984 procedures performed by all survey respondents at the time of completion. 34 surgeons have performed 500 or more endoscopic spine surgeries. During the last 3 months, an average of 24 spine surgeries have been performed per respondent.

The proposed 2-dimensional classification system was determined to be well-suited by 96 respondents (85.7%), while 11 (9.8%) of the respondents considered the classification system to not be well-suited and 5 respondents (4.5%) did not provide an answer to this question. Of the 11 respondents criticizing the classification system, 2 chose “A 2-dimensional classification system is too complicated”, 8 chose “Other parameters should be considered as well,” and 1 chose “Other reason,” and proposed to stage the treatment to pain generator components. Another respondent separately proposed the inclusion of complication rates.

Table 1 provides the results of the evaluation of the technical complexity of the different surgical techniques, as well as the personal experience of the respondents with each technique. Consensus was reached in 12 of the 14 technical procedures. In TELD and IE-LRD, no consensus was reached among the general population of the respondents, prompting additional evaluation of the responses provided by high-volume surgeons with more than 500 endoscopic spine surgeries. For the 12 techniques with consensus among all respondents, the analysis of the subgroup of surgeons with more than 500 endoscopic spine surgeries also agreed with the final results.

**Table 1:** Technical complexity: number of responses and percentage of responses for the technical complexity (grade I – III) and the report of personal experience with each technical procedure

	Evaluation of complexity					Personal experience					
	Grade I	Grade II	Grade III	N/A	Missing	None, np	None, pa	1–5 times	6–50 times	>50 times	Missing
**AECD**	5 (4.5%)	25 (22.3%)	**53 (47.3%)**	29 (25.9%)	0 (0%)	71 (63.4%)	29 (25.9%)	5 (4.5%)	3 (2.7%)	3 (2.7%)	1 (0.9%)
**PECF**	11 (9.8%)	**65 (58%)**	30 (26.8%)	6 (5.4%)	0 (0%)	9 (8%)	39 (34.8%)	25 (22.3%)	27 (24.1%)	11 (9.8%)	1 (0.9%)
**PECCD**	8 (7.1%)	33 (29.5%)	**64 (57.1%)**	7 (6.3%)	0 (0%)	13 (11.6%)	47 (42%)	30 (26.8%)	16 (14.3%)	6 (5.4%)	0 (0%)
**TE-ULBD**	4 (3.6%)	33 (29.5%)	**61 (54.5%)**	14 (12.5%)	0 (0%)	18 (16.1%)	53 (47.3%)	29 (25.9%)	9 (8%)	3 (2.7%)	0 (0%)
**TETD**	10 (8.9%)	30 (26.8%)	**60 (53.6%)**	12 (10.7%)	0 (0%)	18 (16.1%)	46 (41.1%)	28 (25%)	18 (16.1%)	2 (1.8%)	0 (0%)
**TELD**	55 (49.1%)*	49 (43.8%)	7 (6.3%)	1 (0.9%)	0 (0%)	3 (2.7%)	9 (8%)	20 (17.9%)	37 (33%)	42 (37.5%)	1 (0.9%)
**IELD**	**80 (71.4%)**	25 (22.3%)	6 (5.4%)	1 (0.9%)	0 (0%)	4 (3.6%)	7 (6.3%)	16 (14.3%)	31 (27.7%)	54 (48.2%)	0 (0%)
**EELD**	34 (30.4%)	**67 (59.8%)**	10 (8.9%)	1 (0.9%)	0 (0%)	5 (4.5%)	14 (12.5%)	26 (23.2%)	42 (37.5%)	25 (22.3%)	0 (0%)
**TE-LRD**	21 (18.8%)	**64 (57.1%)**	21 (18.8%)	6 (5.4%)	0 (0%)	13 (11.6%)	21 (18.8%)	28 (25%)	36 (32.1%)	13 (11.6%)	1 (0.9%)
**IE-LRD**	45 (40.2%)*	54 (48.2%)	9 (8%)	4 (3.6%)	0 (0%)	5 (4.5%)	11 (9.8%)	24 (21.4%)	40 (35.7%)	32 (28.6%)	0 (0%)
**TELF**	25 (22.3%)	**65 (58%)**	20 (17.9%)	2 (1.8%)	0 (0%)	5 (4.5%)	26 (23.2%)	25 (22.3%)	33 (29.5%)	23 (20.5%)	0 (0%)
**ICELF**	13 (11.6%)	**57 (50.9%)**	36 (32.1%)	6 (5.4%)	0 (0%)	8 (7.1%)	33 (29.5%)	29 (25.9%)	32 (28.6%)	9 (8%)	1 (0.9%)
**LE-ULBD**	19 (17%)	**65 (58%)**	25 (22.3%)	3 (2.7%)	0 (0%)	5 (4.5%)	18 (16.1%)	20 (17.9%)	39 (34.8%)	30 (26.8%)	0 (0%)
**EF**	4 (3.6%)	35 (31.3%)	**63 (56.3%)**	10 (8.9%)	0 (0%)	17 (15.2%)	43 (38.4%)	23 (20.5%)	21 (18.8%)	7 (6.3%)	1 (0.9%)

The given complexity grade is indicated in bold font. The addition of an asterisk (*) indicates no consensus in the general survey respondent population (less than 10% difference between the most common responses) and thus the definition of the complexity grade was based on the responses of high-volume (>500 endoscopic spine surgeries in career) surgeons. (Key: N/*A*, not applicable; np, not planned to employ this technique; pa, potential application of this technique in appropriate cases; AECD, anterior endoscopic cervical discectomy; PECF, posterior endoscopic cervical foraminotomy; PECCD, posterior endoscopic cervical central decompression; TE-ULBD, thoracic endoscopic unilateral laminotomy for bilateral decompression; TETD, transforaminal thoracic decompression/discectomy; TELD, transforaminal endoscopic lumbar discectomy; IELD, interlaminar endoscopic lumbar discectomy; EELD, extraforaminal endoscopic lumbar discectomy; TE-LRD, transforaminal endoscopic lateral recess decompressions; IE-LRD, interlaminar endoscopic lateral recess decompressions; TELF, transforaminal endoscopic lumbar foraminotomies; ICELF, interlaminar contralateral endoscopic lumbar foraminotomies; LE-ULBD, lumbar endoscopic unilateral laminotomy for bilateral decompression; EF, endoscopic fusion (EF)).

97 respondents considered the list of surgical techniques to be complete, while 1 respondent did not provide an answer, and 14 respondents suggested additional entries. These entries included endoscopic rhizotomy (medial branch transection, grade I) and endoscopic decompression of far lateral disc herniations, far-out syndrome, lumbar synovial cysts, and subarticular and cranially migrated herniated nucleus pulposus. Furthermore, endoscopic closure of thoracic spontaneous cerebrospinal fluid leaks, endoscopic tumor resection (intra- and extradural, grade III), endoscopic tethered cord release, and endoscopic decompression for abscess and discitis (grade II – III) were proposed. It was also proposed to classify revision surgeries, multilevel decompressions (grade III), and multilevel spinal fusion surgeries (grade III) separately, and to differentiate fusion procedures into trans-kambin and interlaminar techniques. More specifically, unilateral biportal endoscopic (UBE), contralateral keyhole endoscopic surgery (CKES), bilateral approach for thoracic (calcified) disc herniation (with myelopathy, grade III) and unilateral biportal endoscopic laminoplasty were all proposed. Of these suggestions, rhizotomy was proposed with the greatest frequency, by 4 total respondents.

Anterior endoscopic cervical discectomy (AECD) was removed from the classification system based on the survey results, as the majority of respondents (63.4%) consider this procedure too dangerous. Based on the proposition of 5 respondents (4.4%), endoscopic medial branch transection was added to the list of procedures, and was graded with a complexity grade I.

The results of the evaluation of the added complexity due to morphological characteristics are provided in Table 2. Consensus on the complexity grade was reached for all 9 morphological characteristics. 95 surgeons considered the proposed list to be complete, 1 did not provide an answer to this question, and 16 suggested additional factors. These factors included: severe spinal (c) and foraminal (c) stenosis, the presence of facet joint cysts, and spinal cord involvement (myelopathy, c). It was further suggested to include bony metastasis (b), intra- (c) as well as extradural tumors, the location of migrated disc material, intervertebral disc morphology, small kambin windows, and ossification of ligamentum flavum. Suggested patient-specific factors included body habitus, shoulder height interfering with level counting, scoliosis, and failed back syndrome. The complexity grades were not provided for all suggestions.

**Table 2:** Morphological complexity: number and percentage of responses for the morphological complexity (grade a – c).

	Grade a	Grade b	Grade c	N/A	Missing
**Easy access (eg, interlaminar L5/S1)**	**98 (87.5%)**	11 (9.8%)	1 (0.9%)	1 (0.9%)	2 (1.8%)
**Spinal level adding difficulties (eg, transforaminal approach to L5/S1)**	16 (14.3%)	**73 (65.2%)**	20 (17.9%)	2 (1.8%)	3 (2.7%)
**Potential danger to very critical structures (eg, vertebral artery looping)**	15 (13.4%)	29 (25.9%)	**63 (56.3%)**	4 (3.6%)	5 (4.5%)
**Soft disc herniation**	**100 (89.3%)**	9 (8%)	1 (0.9%)	1 (0.9%)	2 (1.8%)
**Relevant spondylolisthesis / deformity**	11 (9.8%)	**64 (57.1%)**	34 (30.4%)	2 (1.8%)	3 (2.7%)
**Severe degenerative changes**	9 (8%)	**60 (53.6%)**	41 (36.6%)	1 (0.9%)	2 (1.8%)
**Scaring (eg, revision at the same side)**	5 (4.5%)	44 (39.3%)	**59 (52.7%)**	2 (1.8%)	4 (3.6%)
**Calcification of disc herniation**	10 (8.9%)	**66 (58.9%)**	35 (31.3%)	0 (0%)	1 (0.9%)
**Osseous/osteodiscal stenosis**	12 (10.7%)	**69 (61.6%)**	30 (26.8%)	0 (0%)	1 (0.9%)

The given complexity grade is indicated in bold font.

N/*A,* not applicable.

Regarding training for endoscopic spine surgery, simulation training was considered highly effective by 43 respondents, partially effective by 65 respondents, and noneffective by 3 respondents (1 missing entry). Training with cadaveric specimens was considered highly effective by 79 respondents, partially effective by 28 respondents, and noneffective by 3 respondents (2 missing entries). A mean of 26 grade Ia endoscopic spine surgeries (min: 1, max: 100, std: 19.7) was suggested before advancing to more complex surgeries (grade Ib or IIa). To advance from grade Ib or IIa to more complex cases, a mean of 37.1 surgeries were proposed (min: 1, max: 150, std: 32.7).

## Discussion

**Fig. 2:**
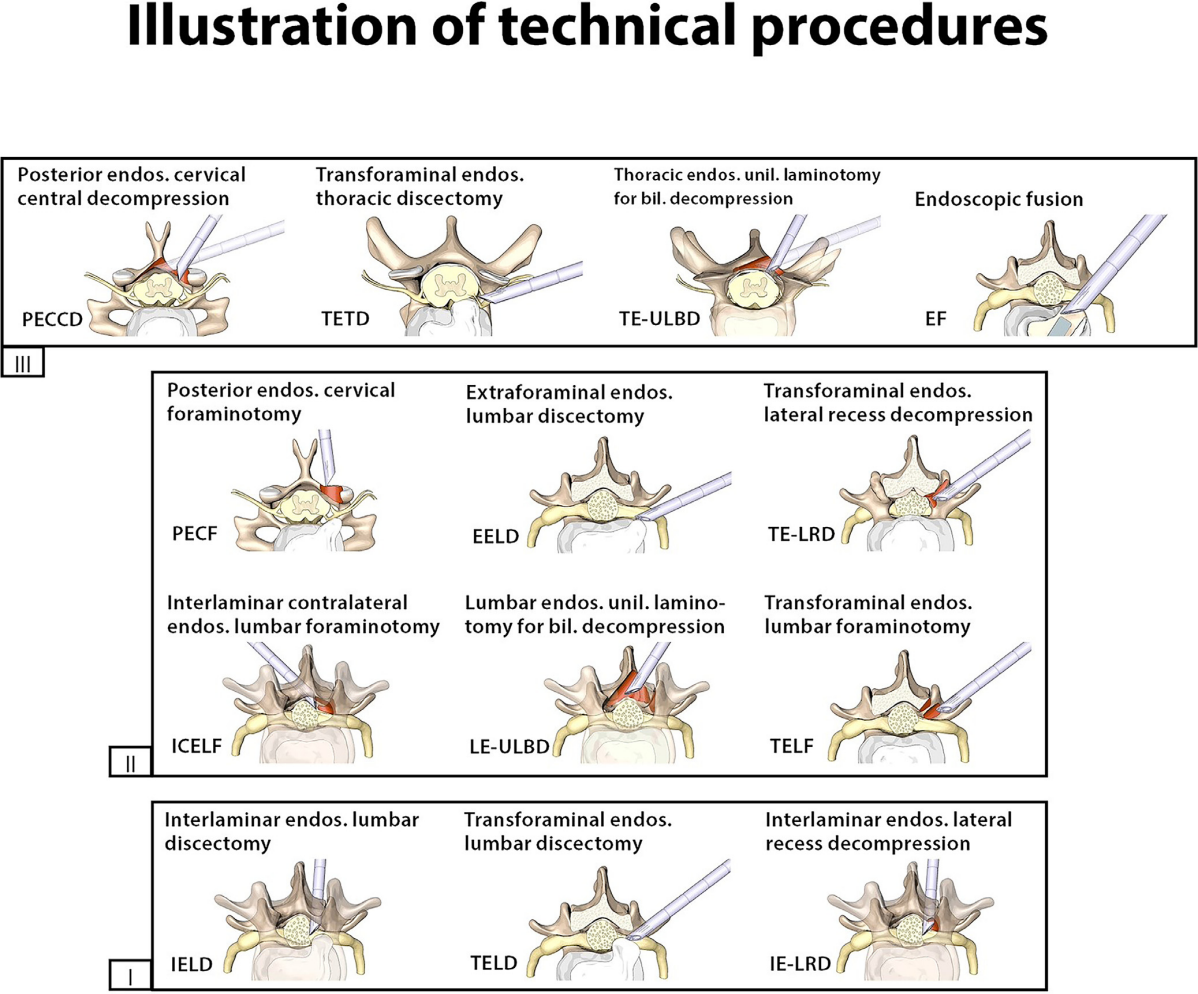
Illustration of the technical procedures grouped according to their complexity from lowest difficulty (grade I) to highest complexity (grade III).

**Fig. 3:**
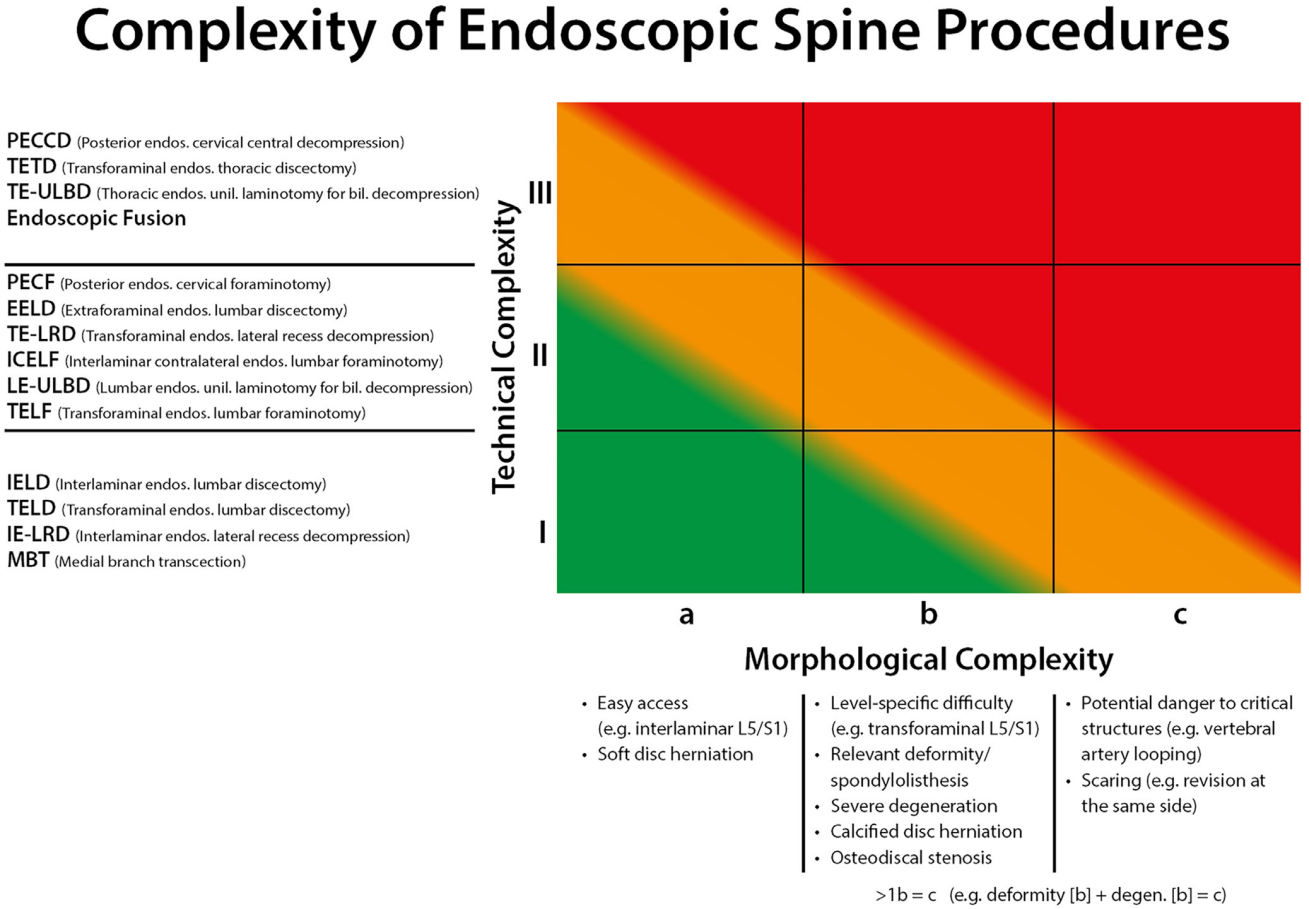
Two-dimensional grading system for the complexity of endoscopic spine procedures with a graded list of technical procedures (vertical, y axis) and morphological parameters (horizontal, x axis).

Endoscopic spine surgery is an emerging technique for minimally invasive spine surgery [[15], [16], [17], [18]], but so far it lacks much structure on categorizing the complexity of differing procedures. Therefore, a classification system - based on technical and morphological complexities - was constructed by including the opinions of more than 1 hundred spine surgeons from 27 countries. The survey results allowed for the creation of agreed-upon nomenclature to classify different technical endoscopic spine procedures into classes of complexity (Fig. 2). Once combined with the consolidated results of the specific morphological characteristics of the pathology to be treated, a more comprehensive and specific classification/complexity scoring algorithm was created (Fig. 3).

The findings and thus justification of Fig. 3 as a classification remain equal when only considering the consolidated results of the 34 experienced surgeons with more than 500 endoscopic spine surgeries. While the authors believe that this classification system will be especially helpful in medical training and implementation of endoscopic spine surgery programs, it should also be useful in both patient education and administrative realms including reimbursement discussions. Further, it will serve as a baseline for communication between endoscopic spine surgeons and for research purposes.

This classification system can serve as a structure for those who want to implement endoscopy of the spine into their practice in 2 ways. First, by recognition of the diversity and hierarchy of endoscopic spine surgery, and following a path starting with lower complexity and progressing towards greater complexity (eg, a beginner should start with a “Ia” procedure such as a soft disc herniation at L5/S1, with a large interlaminar window and without relevant degeneration or scarring, and should not proceed to Ib or IIa procedures until Ia level competence has been achieved. In this survey, an average of 26 endoscopic spine surgeries of grade Ia were suggested before advancing to more complex surgeries (grade Ib or IIa). For advancing from grade Ib or IIa surgeries to even more complex cases, a mean of 37 surgeries were proposed by those surveyed. Although empiric, the suggested numbers are plausible considering reported learning curves investigating the asymptote of such (eg, 35 cases for TELD as described by Yang et al. [19] or Morgenstern R et al. [20], for the first phase of asymptote of the learning curve). Second, implementation of spinal endoscopy into larger institutions with several surgeons could be done using the presented classification. With this classification, the chiefs of spine services receive a tool for a structured practice of spinal endoscopy at their institutions; not only for training, but also for quality control and ensuring excellent patient outcomes.

Further, the classification will improve patient education. Due to the minimally-invasive nature of spinal endoscopy, patients often believe that such procedures might bare smaller risk than traditional spine surgery, and may not differentiate between the wide variety of endoscopic techniques. It is of outmost importance to declare to the patient that, for example, the complexity of a IIIb procedure is much different than than of a Ib procedure. Even if further research is needed to ultimately correlate this classification system with incidence and severity of associated complications, it could certainly serve as grid for patient education in its current state.

In a broader sense, the classification system could also provide some objectivity to health technology assessments and discussions on reimbursements. Choi et al. have found that endoscopic discectomy can save an additional net of $8,064 per QALY [21], but to date, it is unknown if endoscopic procedures of higher complexity would maintain or even possibly improve upon this cost-effectiveness [10]. Even though the authors are unaware of any existent data on the topic, it is plausible to suggest that endoscopic resection of a calcified thoracic disc herniation would be more cost-effective than a thoracic corpectomy and fusion. However, likely in part due to the nascency of endoscopy, in many settings these more complicated endoscopic spinal procedures are not differentiated from their simpler counterparts procedures when considering reimbursement. This classification system could serve as a guide in this regard.

Certainly, the presented classification system has limitations and needs validation for several aspects. The classification represents results of a survey and is therefore built on plausibility and expert opinions and not yet validated regarding patient outcomes or other quantitative data. Surveys are inherently limited in that the answers provided are self-reported and thus cannot be truly verified, as there is no way to confirm or deny for example the exact numbers of operative cases completed by each respondent. However, a large number of surgeons from 27 countries have contributed with their expert opinions, and thus the authors believe that the consolidated results are representative of a comprehensive consensus with a limited risk for biases.

Despite efforts to distribute the survey worldwide, most respondents were from Western countries, resulting in a relative under-representation of Asian spine surgeons, especially considering the vast experience in endoscopic spine surgery in certain Asian countries. Although the data showed no systematic differences between respondents from different countries, it remains unknown whether the classification can be generalized to all regions of the world. Certainly, further research is needed to investigate if the different grades have any impact on surgical factors such as operating time, complications, and clinical outcomes. Also, it remains to be seen if the classification system can serve as a guide for cost-effectiveness in endoscopic spine surgery. Although monoportal and biportal endoscopy share some similarities, the classification presented here focuses on monoportal endoscopy and the validity for biportal techniques needs to be assessed in further studies.

## Conclusion

Based on opinions from more than 1 hundred international spine surgeons from 27 countries, a comprehensive, 2-dimensional classification system for endoscopic spinal procedures has been created, providing instruction for surgical training, implementation of endoscopy in clinical practice, assisting in patient education, and more.

## Funding

There was no external funding for this project.

## Declaration of competing interest

The authors declare that they have no known competing financial interests or personal relationships that could have appeared to influence the work reported in this paper.

## Appendix Supplementary materials

Image, application 1
