# Supplementary material for: Classification of endoscopic spine procedures
Source: N Am Spine Soc J. 2025 Mar 11;22:100603. doi: 10.1016/j.xnsj.2025.100603 (PMC12002952; doi:10.1016/j.xnsj.2025.100603)
Supplement: Supplementary file 1 [file mmc1.pdf]

Complexity of endoscopic spine surgery

Thank you for taking part in this survey, which will only take a few minutes to complete. The data will be anonymised and statistically analysed to develop a grading system for the complexity of endoscopic spine surgery. You may choose to provide your name and email address, which will allow us to validate your responses. Your personal information will not be shared.

Demographic Questions

In which country do you currently practice medicine? __________________________________

In which type of institution do you work (primarily)?
- ◯ University Hospital
- ◯ Hospital
- ◯ Private practice
- ◯ other (please specify)

Please describe your current institution __________________________________

What is your current role?
- ◯ Self employed
- ◯ Chief
- ◯ Consultant
- ◯ Resident
- ◯ Other (please specify)

Please describe your current role __________________________________

What is your current age? __________________________________

Which gender do you identify with?
- ◯ Female
- ◯ Male
- ◯ Other
- ◯ Not reported

Surgical experience

What is your medical specialty?
- ◯ Orthopaedic surgeon
- ◯ Neurosurgeon
- ◯ Other (please specify)

Please describe your medical specialty. __________________________________

How many years have you been performing spine surgery (all surgical techniques)? __________________________________

How many years have you been performing endoscopic spine surgery? __________________________________

What type of endoscopic procedures do you perform?
- ◯ Monoportal
- ◯ Biportal
- ◯ Monoportal and Biportal
- ◯ other (please specify)

Please specify the type of endoscopic procedures you perform

____

In the last 3 months, how many endoscopic procedures have you performed?

____

How many endoscopic procedures have you performed in total?

____

Complexity classification system

We suggest a two-dimensional classification system to quantify the complexity of endoscopic spine surgery considering both technical complexity (the surgical technique), as well as the complexity of morphological conditions (anatomy and characteristics of the pathology):

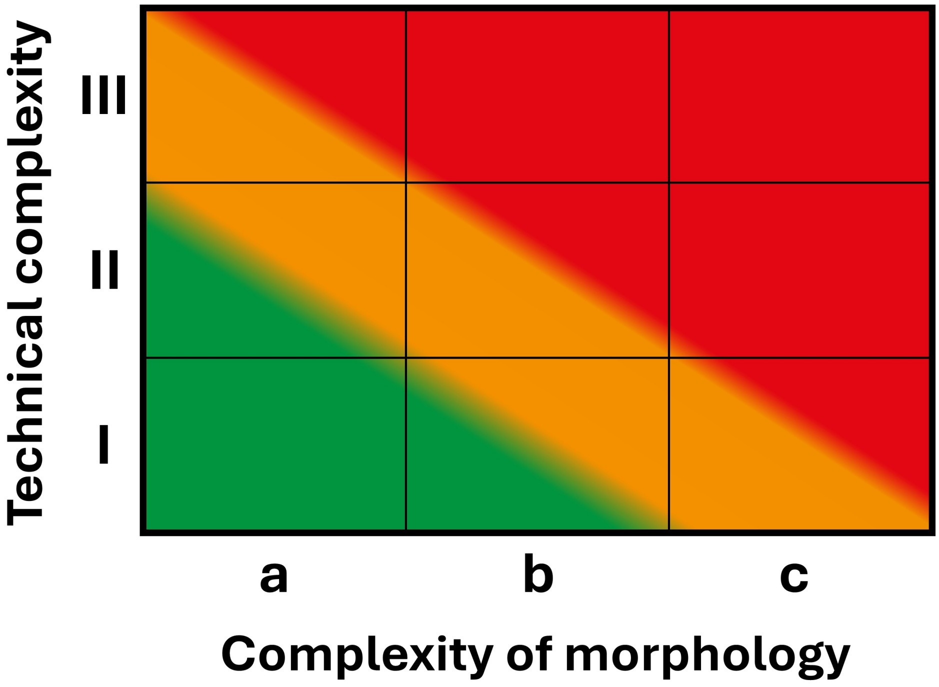

Do you think the proposed classification system is well suited to quantify the complexity of endoscopic spine surgery?

◯ Yes
◯ No

Why do you think the proposed classification system is not well suited (multiple responses possible)?

☐ A two dimensional classification system is too complicated
☐ Other parameters should be considered as well
☐ Other reason (please specify)

Please describe, why you consider the proposed classification system not to be well suited.

____

Please describe your proposed alternative classification system for the complexity of endoscopic spine surgery.

____

Technical Complexity
Please rate the technical complexity of the following endoscopic procedures from the simplest (grade I) to the most complex (grade III). If you don't feel able to rate the complexity, please select NA (not applicable).

Anterior endoscopic cervical discectomy (AECD)

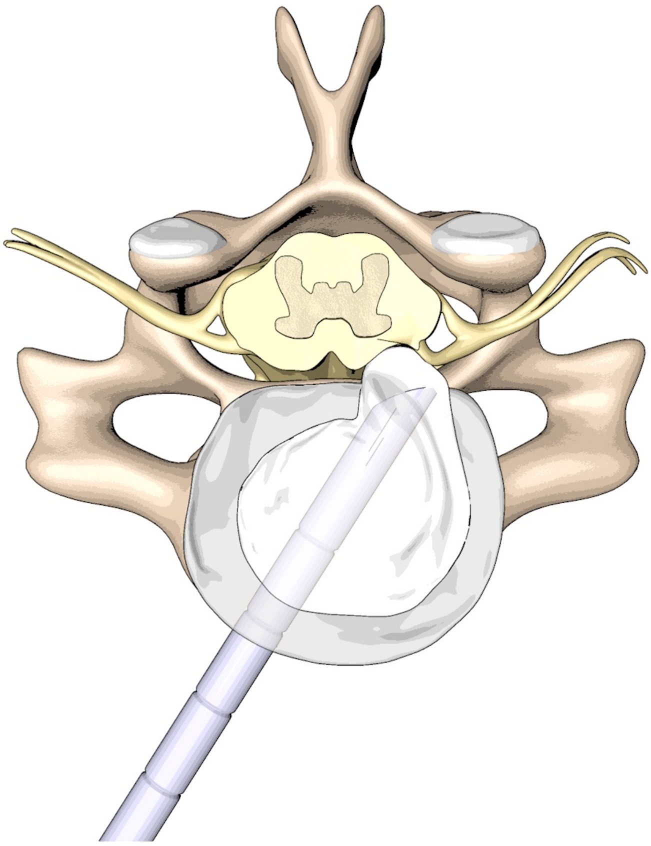

Please rate the complexity of anterior endoscopic cervical discectomy (AECD)	◯ grade I<br>◯ grade II<br>◯ grade III<br>◯ NA
How often have you performed anterior endoscopic cervical discectomy (AECD)?	◯ 0, and I don't plan to use this approach<br>◯ 0, but I will use it in appropriate cases<br>◯ 1 - 5 times<br>◯ 6 - 50 times<br>◯ > 50 times

Posterior endoscopic cervical foraminotomy (PECF)

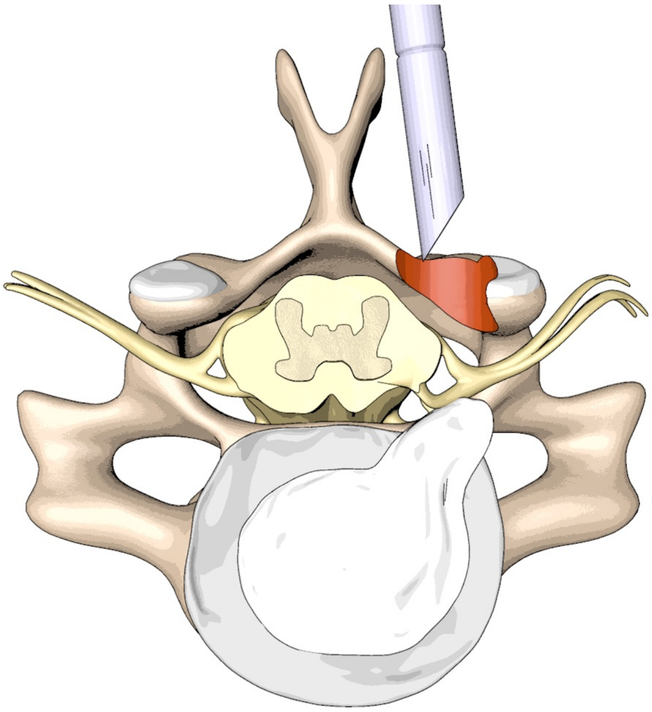

Please rate the complexity of posterior endoscopic cervical foraminotomy (PECF)

- ◯ grade I
- ◯ grade II
- ◯ grade III
- ◯ NA

How often have you performed posterior endoscopic cervical foraminotomy (PECF)?

- ◯ 0, and I don't plan to use this approach
- ◯ 0, but I will use it in appropriate cases
- ◯ 1 - 5 times
- ◯ 6 - 50 times
- ◯ > 50 times

Posterior endoscopic cervical central decompression (PECCD)

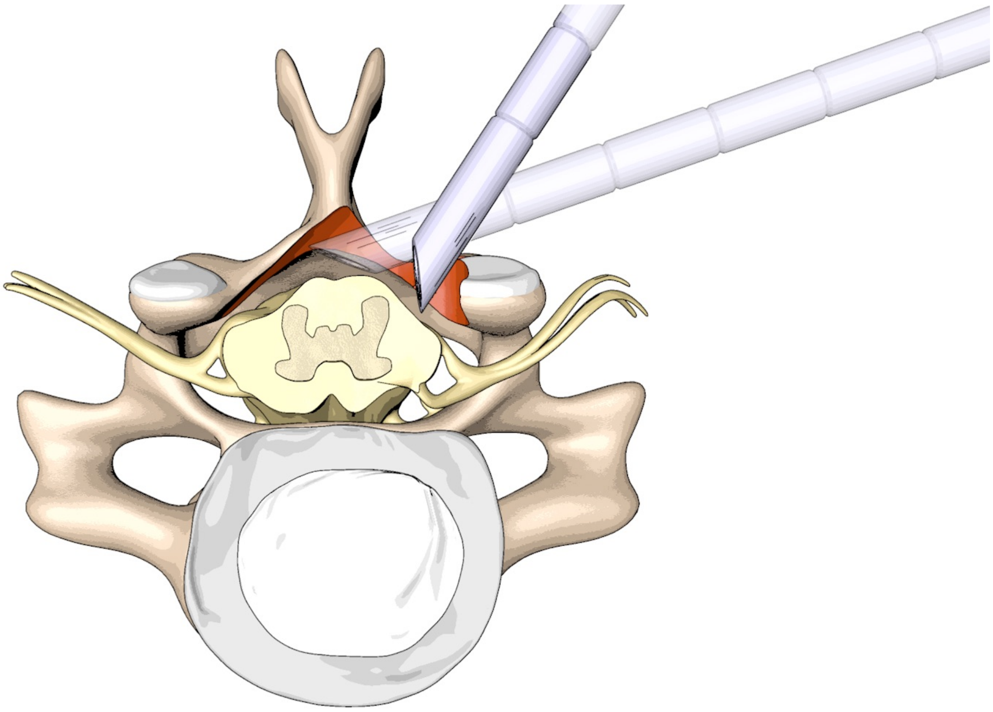

Please rate the complexity of posterior endoscopic cervical central decompression (PECCD)

- ◯ grade I
- ◯ grade II
- ◯ grade III
- ◯ NA

How often have you performed posterior endoscopic cervical central decompression (PECCD)?

- ◯ 0, and I don't plan to use this approach
- ◯ 0, but I will use it in appropriate cases
- ◯ 1 - 5 times
- ◯ 6 - 50 times
- ◯ > 50 times

Thoracic endoscopic unilateral laminotomy for bilateral decompression (TE-ULBD)

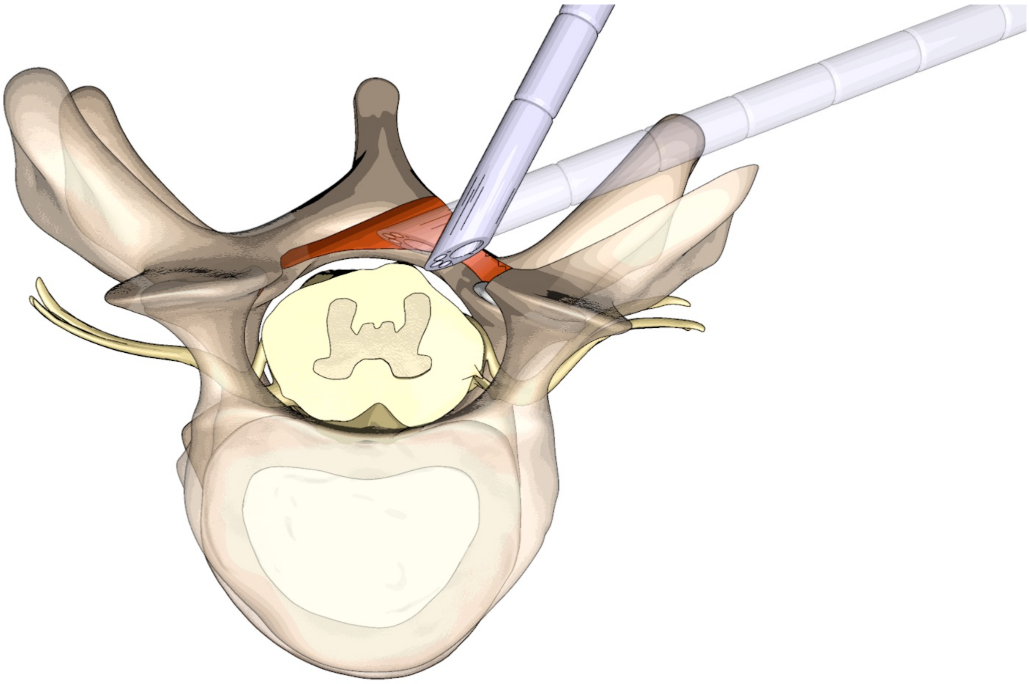

Please rate the complexity of thoracic endoscopic unilateral laminotomy for bilateral decompression (TE-ULBD)	◯ grade I<br>◯ grade II<br>◯ grade III<br>◯ NA
How often have you performed thoracic endoscopic unilateral laminotomy for bilateral decompression (TE-ULBD)?	◯ 0, and I don't plan to use this approach<br>◯ 0, but I will use it in appropriate cases<br>◯ 1 - 5 times<br>◯ 6 - 50 times<br>◯ > 50 times

Transforaminal thoracic decompression/discectomy (TETD)

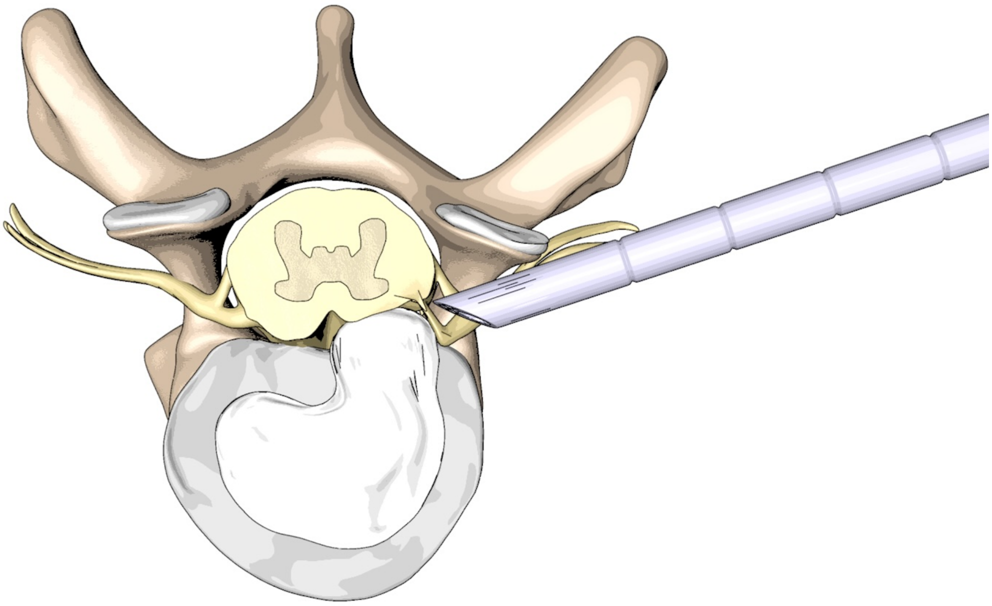

Please rate the complexity of transforaminal thoracic decompression/discectomy (TETD)	◯ grade I<br>◯ grade II<br>◯ grade III<br>◯ NA
How often have you performed transforaminal thoracic decompression/discectomy (TETD)?	◯ 0, and I don't plan to use this approach<br>◯ 0, but I will use it in appropriate cases<br>◯ 1 - 5 times<br>◯ 6 - 50 times<br>◯ > 50 times

Transforaminal endoscopic lumbar discectomy (TELD)

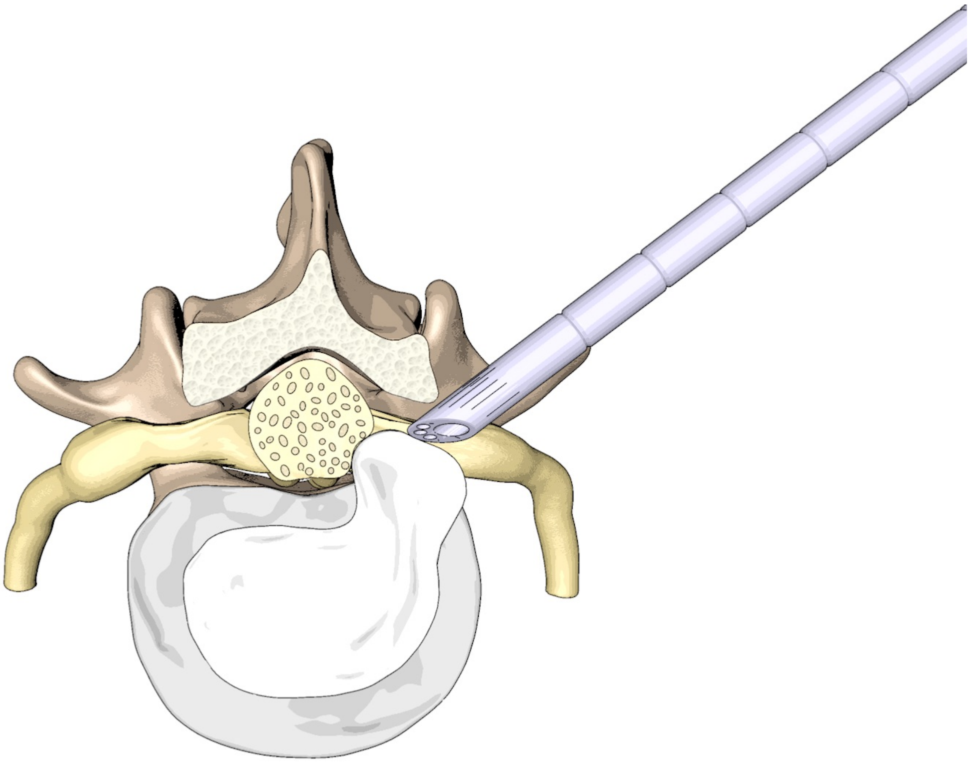

Please rate the complexity of transforaminal endoscopic lumbar discectomy (TELD)

- ◯ grade I
- ◯ grade II
- ◯ grade III
- ◯ NA

How often have you performed transforaminal endoscopic lumbar discectomy (TELD)?

- ◯ 0, and I don't plan to use this approach
- ◯ 0, but I will use it in appropriate cases
- ◯ 1 - 5 times
- ◯ 6 - 50 times
- ◯ > 50 times

Interlaminar endoscopic lumbar discectomy (IELD)

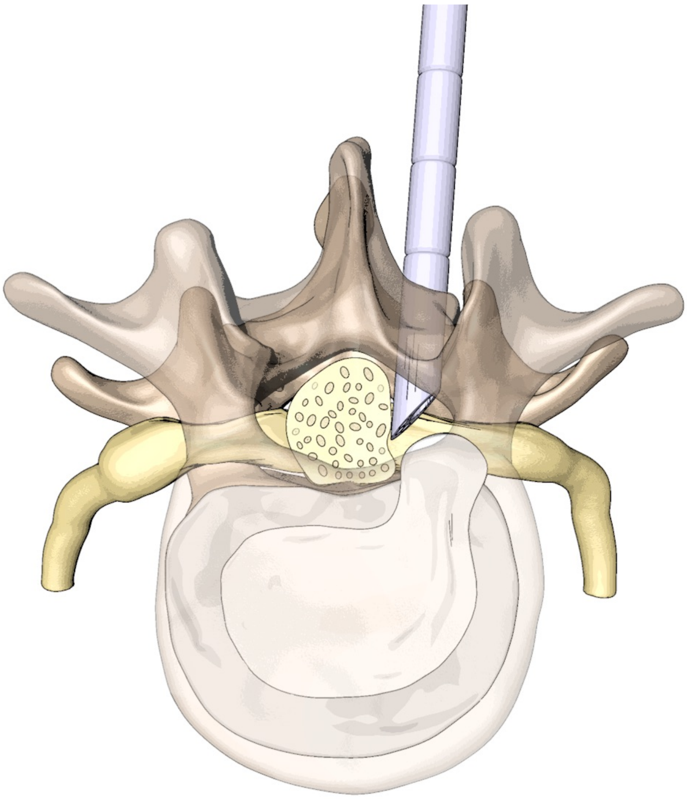

Please rate the complexity of interlaminar endoscopic lumbar discectomy (IELD)	◯ grade I<br>◯ grade II<br>◯ grade III<br>◯ NA
How often have you performed interlaminar endoscopic lumbar discectomy (IELD)?	◯ 0, and I don't plan to use this approach<br>◯ 0, but I will use it in appropriate cases<br>◯ 1 - 5 times<br>◯ 6 - 50 times<br>◯ > 50 times

Extraforaminal endoscopic lumbar discectomy (EELD)

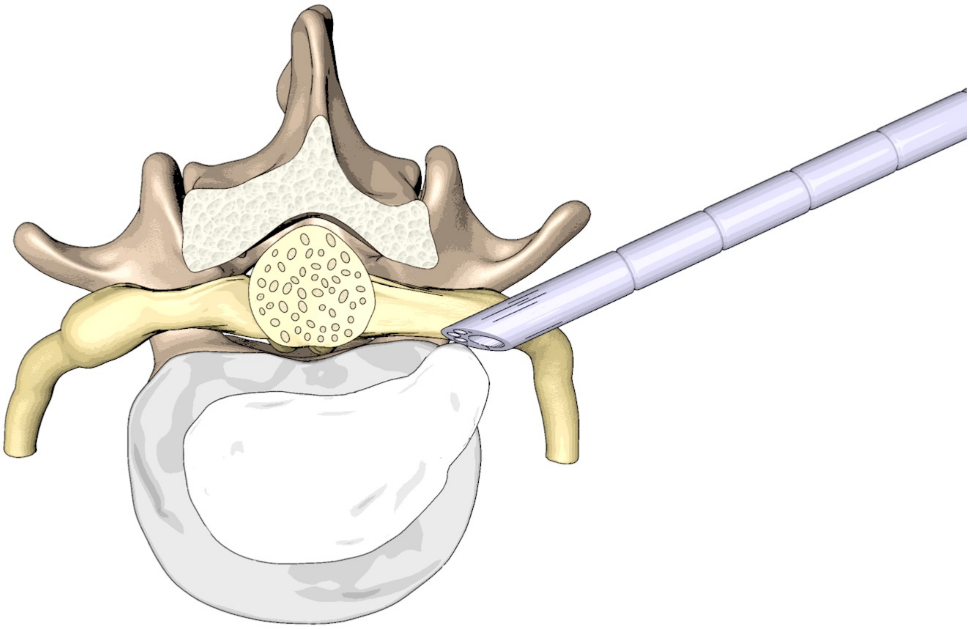

Please rate the complexity of extraforaminal endoscopic lumbar discectomy (EELD)	◯ grade I<br>◯ grade II<br>◯ grade III<br>◯ NA
How often have you performed extraforaminal endoscopic lumbar discectomy (EELD)?	◯ 0, and I don't plan to use this approach<br>◯ 0, but I will use it in appropriate cases<br>◯ 1 - 5 times<br>◯ 6 - 50 times<br>◯ > 50 times

Transforaminal endoscopic lateral recess decompressions (TE-LRD)

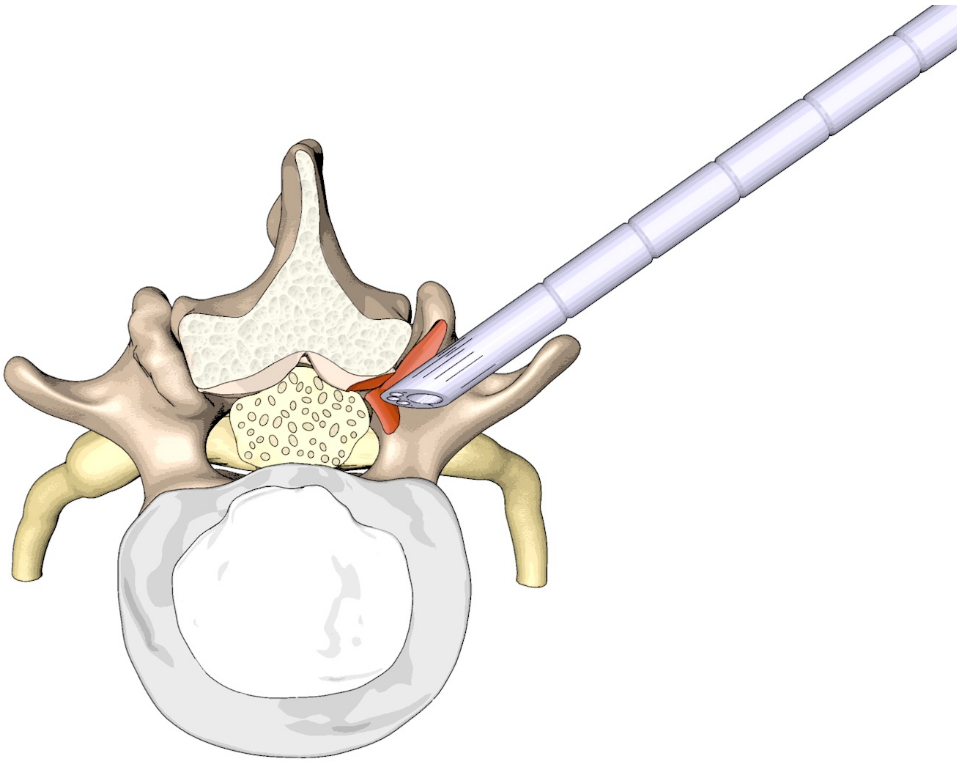

Please rate the complexity of transforaminal endoscopic lateral recess decompressions (TE-LRD)

- ◯ grade I
- ◯ grade II
- ◯ grade III
- ◯ NA

How often have you performed transforaminal endoscopic lateral recess decompressions (TE-LRD)?

- ◯ 0, and I don't plan to use this approach
- ◯ 0, but I will use it in appropriate cases
- ◯ 1 - 5 times
- ◯ 6 - 50 times
- ◯ > 50 times

Interlaminar endoscopic lateral recess decompressions (IE-LRD)

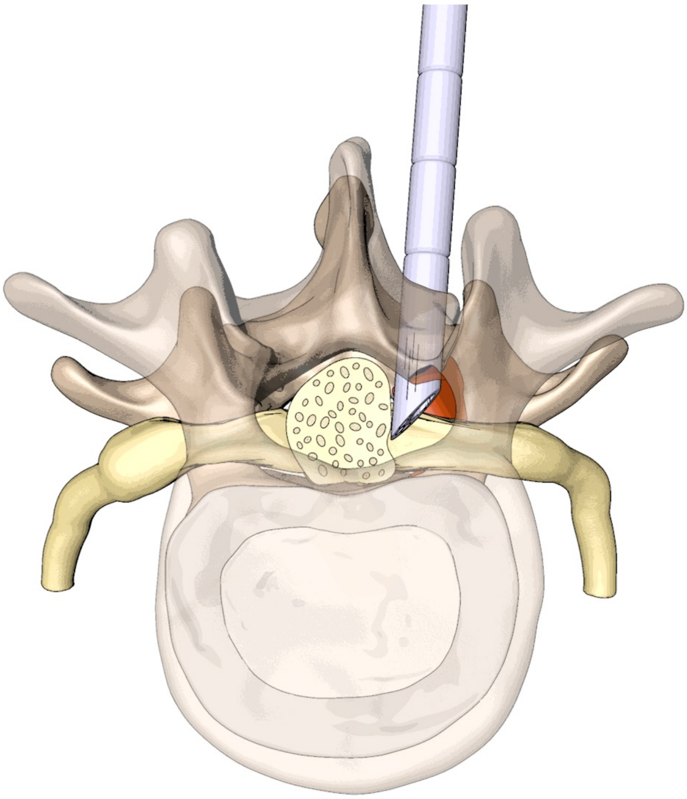

Please rate the complexity of interlaminar endoscopic lateral recess decompressions (IE-LRD)	◯ grade I<br>◯ grade II<br>◯ grade III<br>◯ NA
How often have you performed interlaminar endoscopic lateral recess decompressions (IE-LRD)?	◯ 0, and I don't plan to use this approach<br>◯ 0, but I will use it in appropriate cases<br>◯ 1 - 5 times<br>◯ 6 - 50 times<br>◯ > 50 times

Transforaminal endoscopic lumbar foraminotomies (TELF)

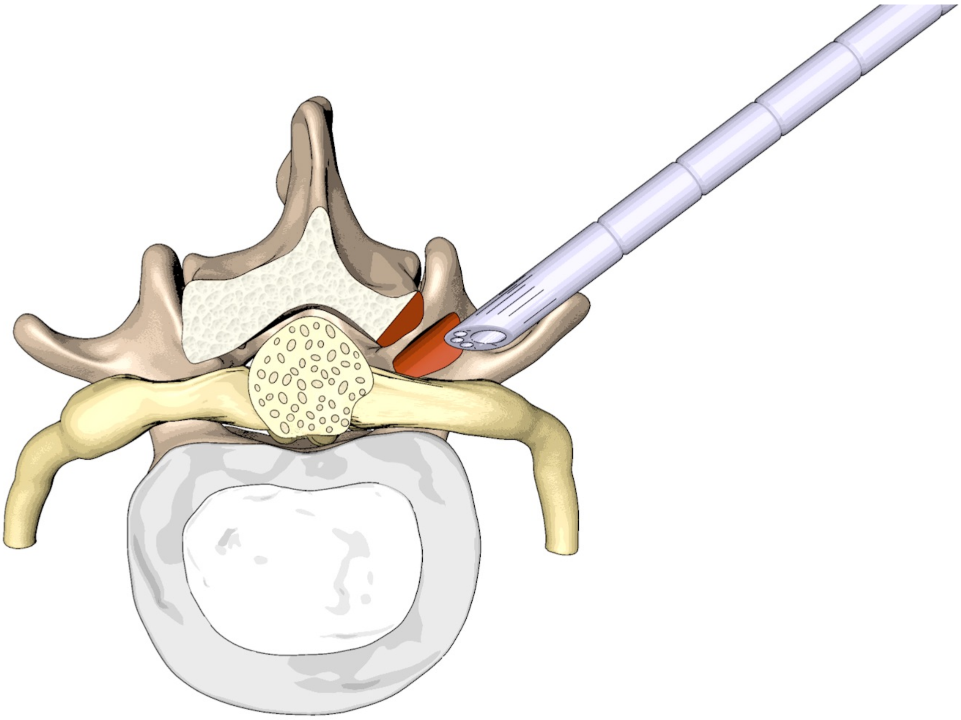

Please rate the complexity of transforaminal endoscopic lumbar foraminotomies (TELF)	◯ grade I<br>◯ grade II<br>◯ grade III<br>◯ NA
How often have you performed transforaminal endoscopic lumbar foraminotomies (TELF)?	◯ 0, and I don't plan to use this approach<br>◯ 0, but I will use it in appropriate cases<br>◯ 1 - 5 times<br>◯ 6 - 50 times<br>◯ > 50 times

Interlaminar contralateral endoscopic lumbar foraminotomies (ICELF)

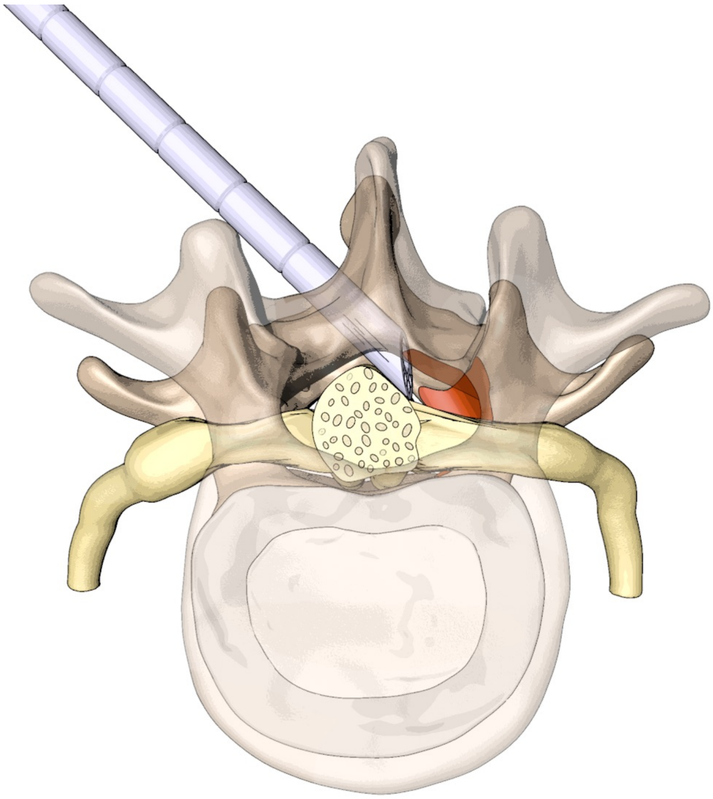

Please rate the complexity of interlaminar contralateral endoscopic lumbar foraminotomies (ICELF)	◯ grade I<br>◯ grade II<br>◯ grade III<br>◯ NA
How often have you performed interlaminar contralateral endoscopic lumbar foraminotomies (ICELF)?	◯ 0, and I don't plan to use this approach<br>◯ 0, but I will use it in appropriate cases<br>◯ 1 - 5 times<br>◯ 6 - 50 times<br>◯ > 50 times

Lumbar endoscopic unilateral laminotomy for bilateral decompression (LE-ULBD)

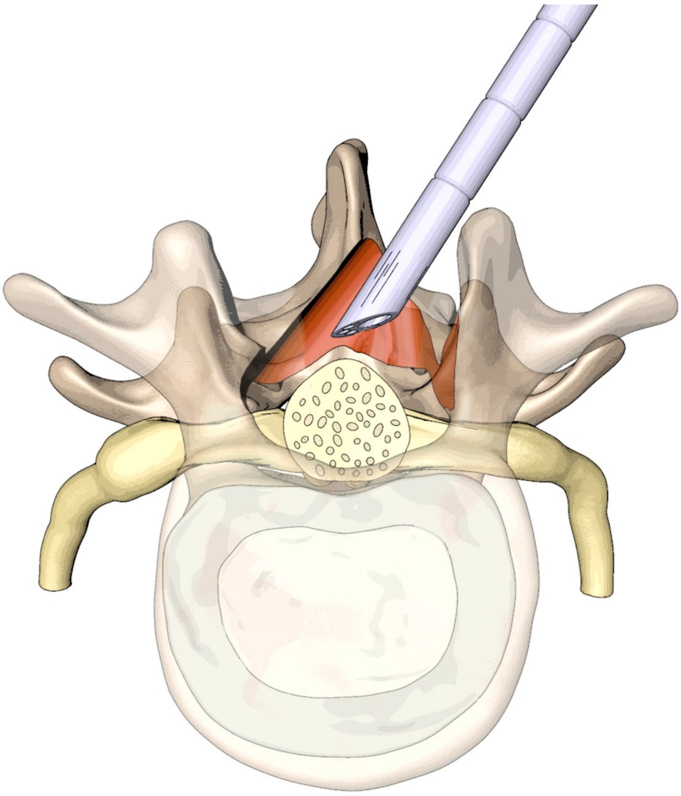

Please rate the complexity of lumbar endoscopic unilateral laminotomy for bilateral decompression (LE-ULBD)	◯ grade I<br>◯ grade II<br>◯ grade III<br>◯ NA
How often have you performed lumbar endoscopic unilateral laminotomy for bilateral decompression (LE-ULBD)?	◯ 0, and I don't plan to use this approach<br>◯ 0, but I will use it in appropriate cases<br>◯ 1 - 5 times<br>◯ 6 - 50 times<br>◯ > 50 times

Endoscopic fusions

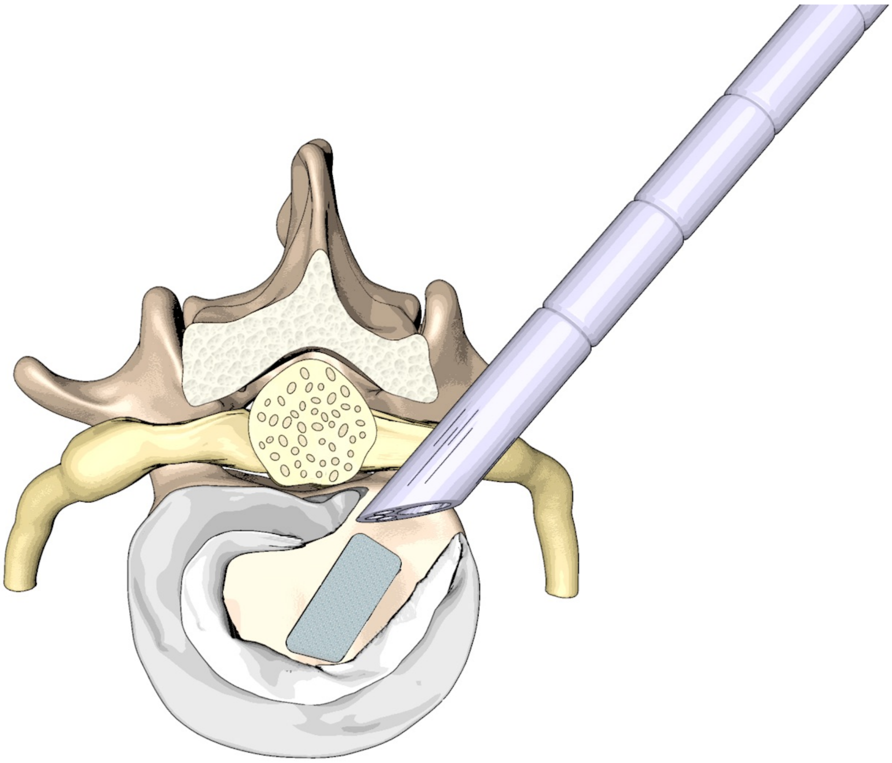

Please rate the complexity of endoscopic fusions	◯ grade I<br>◯ grade II<br>◯ grade III<br>◯ NA
How often have you performed endoscopic fusions?	◯ 0, and I don't plan to use this approach<br>◯ 0, but I will use it in appropriate cases<br>◯ 1 - 5 times<br>◯ 6 - 50 times<br>◯ > 50 times
Do you consider the above list of endoscopic procedures to be complete?	◯ Yes<br>◯ No (please specify)
Please list the missing procedures with the corresponding grade of complexitiy (grade I - III)	__________________________________

Complexity of morphology
Please rate the added complexity of any endoscopic procedure due to below listed morphological parameters and conditions (a = no/minimal change in complexity, b = intermediate increase of complexity, c = large increase of complexity). If you do not feel able to assess the added complexity, select NA (not applicable).

	a	b	c	NA

Easy access (e.g., interlaminar L5/S1)	◯	◯	◯	◯
Spinal level adding difficulties (e.g. transforaminal approach to L5/S1)	◯	◯	◯	◯
Potential danger to very critical structures (e.g. vertebral artery looping)	◯	◯	◯	◯
Soft disc herniation	◯	◯	◯	◯
Relevant spondylolisthesis / deformity	◯	◯	◯	◯
Severe degenerative changes	◯	◯	◯	◯
Scaring (e.g., revision at the same side)	◯	◯	◯	◯
Calcification of disc herniation	◯	◯	◯	◯
Osseous/osteodiscal stenosis	◯	◯	◯	◯

Do you consider the above list of morphological conditions (relevant to complexity) to be complete?

◯ Yes
◯ No (please specify)

Please list the missing morphologic conditions relevant to the complexity with the corresponding grade of complexitiy (grade a, b or c)

____

Training

In your opinion, how many simple (grade Ia) complexity cases should be performed before progressing to more complex cases (e.g., grade Ib or IIa cases)?

____

In your opinion, how many cases with intermediate complexity (e.g., grade IIa or Ib) should be performed before progressing to the most complex cases?

____

Do you think simulation training is an effective training method for endoscopic spine surgery?

◯ highly effective
◯ partially effective
◯ no effectiveness

Do you consider cadaver training to be an effective training method for endoscopic spine surgery?

◯ highly effective
◯ partially effective
◯ no effectiveness

Personal information and feedback

Email address (optional, will not be published) ________________________________

Name (optional, will not be published) ________________________________

Would you like to be informed about the results of this survey? (email address required)
◯ Yes
◯ No

Do you want to add anything regarding the content of this survey? ________________________________
